# Enhancing the accuracy of molecular classification of pediatric CNS tumors: a dual-classifier approach using DNA methylation profiling

**DOI:** 10.3389/fonc.2025.1701113

**Published:** 2026-02-05

**Authors:** Esra Moosa, Rania Alanany, Shimaa Sherif, Erdener Ozer, Sukoluhle Dube, Aayesha Jabeen, Apryl Sanchez, Asma Jamil, Aisha Khalifa, Chiara Cugno, Ian Pople, Davide Bedognetti, Ata Maaz, Ayman Saleh, William Mifsud, Wouter R. L. Hendrickx, Christophe M. Raynaud

**Affiliations:** 1 Anatomical Pathology Division, Department of Clinical Pathology, Sidra Medicine, Doha, Qatar; 2 Department of Biomedical Science, College of Health Sciences, Qatar University, Doha, Qatar; 3 Tumor Biology and Immunology Laboratory (TBI), Sidra Medicine, Doha, Qatar; 4 College of Health and Life Sciences (CHLS), Hamad Bin Khalifa University (HBKU), Doha, Qatar; 5 Clinical Trials Office, Sidra Medicine, Doha, Qatar; 6 Advanced Cell Therapy Core, Research Department, Sidra Medicine, Doha, Qatar; 7 Neurosurgery Division, Sidra Medicine, Doha, Qatar; 8 Clinical and Experimental Oncology and Hematology, Ospedale Policlinico San Martino, Genova, Italy; 9 Oncology and Hematology Division, Sidra Medicine, Doha, Qatar

**Keywords:** CNS tumor classification, FFPE (formalin fixed paraffin embedded), methylation, pediatric cancer, EPIC arrays, remove diagnostic

## Abstract

DNA methylation-based classification has improved central nervous system (CNS) tumor diagnostics, but pediatric data on real-world implementation remain limited. We evaluated two DNA methylation-based classifiers—the Heidelberg classifier and the NIH/Bethesda (Methylscape) classifier—in a single-center cohort of pediatric patients. A total of 96 samples from 96 patients (75 CNS tumors, 10 non-CNS tumors, and 11 non-neoplastic CNS lesions) were profiled using Illumina MethylationEPIC arrays (850K/930K). We compared calibrated scores, concordance with integrated histopathological diagnoses, and the impact of technical factors such as tissue preservation, analyzable CpG count, and array version. Methylation classification agreed with integrated histopathology in 88.0% (66/75) of CNS tumors and refined diagnoses in 54.7% (41/75). Both classifiers showed high concordance but occasionally assigned high-confidence labels to non-neoplastic lesions, underscoring the importance of joint pathological review. Fresh frozen versus FFPE tissue, analyzable CpG count, and EPIC v1 versus v2 did not significantly affect classifier performance in our setting. Our findings support the use of methylation classifiers as decision-support tools in pediatric CNS tumor diagnostics, provided that calibrated score thresholds are interpreted in the context of tumor purity, DNA quality, and integrated neuropathology.

## Introduction

Accurate diagnosis and molecular classification of central nervous system (CNS) tumors are critical for optimal patient management and treatment. Historically, CNS tumor classification and grading relied primarily on histological assessment, which, while essential, is inherently prone to inter-observer variability and experience. Apart from the difficulties in histopathologic diagnosis, including inter-observer variability and differences in pathologists’ experience, the availability and quality of ancillary stains—particularly immunohistochemistry panels tailored to CNS tumor diagnosis—also critically influence diagnostic accuracy. In many settings where advanced molecular assays are not available or affordable, conventional histology diagnosis supported by appropriate immunohistochemistry still remains the main and often sufficient approach for CNS tumor diagnosis. This results in a significant rate of misclassification among pathologists (1). Recognizing the limitations of morphology-based diagnosis, advanced molecular testing has become indispensable, offering critical insights for diagnosis, prognosis, and targeted therapy (2, 3). The World Health Organization (WHO) incorporated molecular analyses alongside histological evaluations in its updated CNS tumor classification systems, first in 2016 and later in 2021, listing numerous genetic alterations critical for diagnosis, including many mutations and copy number variations (4).

Among these molecular advancements, DNA methylation profiling has emerged as a powerful and reliable tool for CNS tumor classification. It has proven especially valuable for diagnosing tumors with challenging or atypical morphology and for identifying biologically distinct subtypes within certain tumor families, such as gliomas, ependymomas, and medulloblastomas—for instance, ependymomas, though morphologically similar, are now classified into distinct biological types associated with specific anatomic locations and patient populations, with marked differences in prognosis and patterns of relapse. Supratentorial ependymoma types are defined by genetic alterations such as ZFTA and YAP1 fusions, while others, such as posterior fossa groups A and B, are most accurately distinguished through DNA methylation profiling (5–9); these molecular groups are increasingly used to stratify patients in risk-adapted clinical trials. Similarly, medulloblastoma subgroups, initially characterized by transcriptome analysis, are now reliably classified using DNA methylation profiling (10–12), and these subgroups directly inform treatment intensity and clinical trial design—for example, recognition of high-risk group 3 medulloblastoma has prompted intensified strategies such as the addition of carboplatin to craniospinal irradiation (13), while the identification and methylation-based delineation of embryonal tumor with multilayered rosettes (ETMR) has enabled focused, biology-driven trials such as the recent PNOC ETMR study (14, 15). These examples illustrate how increased diagnostic resolution translates into concrete changes in therapy, risk stratification, and translational research opportunities.

In addition, DNA methylation profiling has shown promise in refining diagnoses for tumors with overlapping or heterogeneous morphology, such as pediatric diffuse gliomas and embryonal tumors (16–18). Methylation-derived subclasses often correlate with prognosis and therapeutic response, providing clinically meaningful stratification beyond conventional histopathology (19, 20).

To capitalize on tumor-specific epigenetic signatures, a machine learning-based DNA methylation classifier was developed by the German Cancer Research Center and Heidelberg University (commonly referred to as the “Heidelberg classifier”) and made freely accessible as a research tool via an online portal (21). Although initially designed for research purposes, DNA methylation profiling has since been successfully implemented in the clinical setting at several institutions—including the NIH, St. Jude Children’s Research Hospital, Northwestern University, NYU, and Princess Máxima Center for Pediatric Oncology—demonstrating compliance with local regulatory or accreditation frameworks, including but not limited to the College of American Pathologists (CAP) (22–25). Despite this growing adoption, broader integration into clinical workflows remains limited by technical, regulatory, and logistical barriers, particularly across varied tissue types and platforms.

Previous studies have explored alternative classification models using pediatric CNS tumor cohorts, including Tran et al. (25), who compared three distinct informatic approaches—random forest, support vector machine, and multiclass perceptron neural network—trained on the Capper et al. dataset. However, these models were custom-built, not publicly available, and require substantial bioinformatics expertise to implement. To our knowledge, few studies directly compare the Heidelberg and NIH classifiers within a single pediatric CNS cohort; here we provide a side-by-side evaluation with unified endpoints. Additionally, we directly compare the performance of Illumina MethylationEPIC v1 and v2 arrays using both formalin-fixed paraffin-embedded (FFPE) and fresh frozen (FF) tissues. Finally, we assess how technical variables—including DNA input quantity and fixation method—impact the quality and reliability of classification, with the goal of informing broader implementation in routine clinical diagnostics.

## Materials and methods

### Sample collection

This retrospective study included 96 pediatric patients diagnosed and/or treated at Sidra Medicine, Qatar, between January 2018 and December 2024. As summarized in **Table 1**, the cohort comprised 75 CNS tumors, 10 non-CNS tumors, and 11 non-neoplastic CNS lesions. For all cases, diagnostic hematoxylin and eosin (H&E)-stained slides were reviewed by the neuropathology team.

**Table 1:** List of patients and lesion characteristics.

All patients		
Male		54
Female		42
Mean age at diagnosis (years)		6.92
Lesion categories		
CNS tumor, *N* = 75		
Male		43
Female		32
Mean age at diagnosis (in years)		6.61
Gliomas	• High-grade gliomas, NOS (*n* = 7) • Glioblastoma (*n* = 1) • Low-grade gliomas, NOS (*n* = 2) • Pilocytic astrocytoma (*n* = 18) • Pleomorphic xanthoastrocytoma (*n* = 3) • Subependymal giant cell astrocytoma (*n* = 1)	32
Embryonal tumors	• Medulloblastoma (*n* = 17) • ATRT (*n* = 4) • Ependymoblastoma (*n* = 1)	22
Glioneuronal tumor	• Glioneuronal tumor, NOS (*n* = 2) • Ganglioglioma (*n* = 1) • Dysembryoplastic neuroepithelial tumor (*n* = 2)	5
Ependymoma		7
Germ cell tumors		3
Pineoblastoma		3
Choroid plexus papilloma		1
Craniopharyngioma		1
Meningioma		1
CNS non-tumor, *N* = 10		
Male		4
Female		6
Mean age at diagnosis (in years)		7.9
CNS non-tumor	• Vascular malformation (*n* = 3) • Epilepsy associated lesions (*n* = 6) • Malformative lesion (*n* = 1)	10
Non-CNS tumors, *N* = 11		
Male		7
Female		4
Mean age at diagnosis (in years)		8
Non-CNS tumors	• Germ cell tumor (*n* = 6) • Neuroblastoma (*n* = 2) • Rhabdomyosarcoma (*n* = 1) • Synovial sarcoma (*n* = 1) • Undifferentiated sarcoma (*n* = 1)	11

### DNA extraction

DNA was extracted from formalin-fixed paraffin-embedded (FFPE) tissue blocks or fresh frozen (FF) samples. In total, 96 samples from 96 patients were available for DNA extraction, including CNS tumors, non-CNS tumors, and non-neoplastic CNS lesions. When multiple blocks were available for a case, the block with the highest tumor content was selected based on H&E review.

DNA extraction was performed using either the DNeasy Blood and Tissue Kit (#69504, Qiagen) or the Maxwell^®^ RSC Tissue DNA Kit (AS1610, Promega) on the Maxwell RSC instrument (AS4500, Promega) following the manufacturers’ protocols.

Two pathologists (W.M. and E.O.) and one research specialist (C.R.) estimated the number of tumor cells in each sample case. The tumor cell content was assessed based on the H&E-stained slides. The H&E slides were cut and pre-checked before submitting the FFPE tissue blocks for DNA extraction and methylation array analysis. DNA was extracted from six 10-µm-thickness curls, either immediately or after storage at -80 °C for extraction on the following day, using the Maxwell^®^ RSC DNA FFPE Kit (#AS1450, Promega).

DNA quantification was performed using the Quantus Fluorometer (E6150, Promega) with the QuantiFluor^®^ ONE dsDNA System (#E4870, Promega). We aimed to use 500 ng of DNA in 12 µL per sample or the highest possible concentration in up to 35 µL. Bisulfite conversion was carried out using the EZ DNA Methylation™ Kit (#D5002, Zymo Research). Samples with DNA concentrations below 8.5 ng/µL after extraction were excluded (less than 300 ng for methylation array analysis).

### Methylation analysis

In total, we performed 125 methylation array runs. Two samples were analyzed three times as experimental controls, and one patient’s DNA samples (from two extractions) were analyzed 17 times as a control in the methylation profiling experiments (three from one extraction and 14 from the second extraction of the same FFPE block).

The DNA sample used as a control in each batch of our analyses served to monitor both the experiment and the technical aspects of the procedure. It was specifically used to ensure that for every batch of eight to 46 samples processed at once, the procedures ran smoothly and no technical issues arose, thus validating the accuracy and reliability of the results obtained from each batch.

Methylation data were generated using either the Illumina MethylationEPIC (850k) BeadChip platform (v1) for 40 samples or the Infinium MethylationEPIC v2.0 Kit (930k) (v2) for the remaining samples, following the methodology described by Capper et al. (1) and the manufacturer’s recommendations.

For each sample, paired IDAT files (red and green) were uploaded into the Heidelberg DNA Methylation Tumor Classifier (version 12.8, www.molecularneuropathology.org) or in the methylscape analysis profiler from NIH (https://methylscape.ccr.cancer.gov/).

The Heidelberg classifier assigned, when possible, a methylation “superfamily” and, if applicable, a more specific “family”, “class”, or “subclass” within that superfamily, along with a calibrated score ranging from 0 to 0.99. This score indicates the degree of similarity between the sample’s methylation profile and the reference database. The scores above 0.9 are considered a match, while scores between 0.9 and 0.3 are considered as no match but indicative as they could still be relevant for cases with low tumor content or poor DNA quality. Scores below 0.3 are considered a “no match”. We personally introduced another category with scores between 0.9 and 0.84, as the useful confidence limit was mentioned to be possibly as low as 0.84 by Capper et al. (21).

With the NIH methylscape classifier, when possible, a methylation “family” and, if applicable, a more specific “class “are provided along with a calibrated score ranging from 0 to 0.99. Scores above 0.9 are considered a match, while scores between 0.9 and 0.5 are considered indicative. Scores below 0.5 are considered a “no match”. The NIH methylscape classifier also provides a location of the specific analyzed specimen within a UMAP of the samples used for classifier calibration, as interpretation can often be improved with visual inspection of unsupervised UMAP embedding.

### t-SNE analysis

Raw signal intensities were extracted from IDAT files using the minfi Bioconductor package (v.11.52.1). Samples were normalized by background correction and dye-bias correction via the function of the funnorm. Beta values, obtained using the getBeta function, ranged from 0 (completely unmethylated) to 1 (completely methylated). For betas derived from the epic_v2 dataset, betasCollapseToPfx function from the sesame package (v.1.24.0) was used to collapse the beta values by averaging probes with a common probe ID prefix. Since no batch effects were detected, no batch correction was done. During the filtration process, the following probes were removed: (a) probes targeting the X and Y chromosomes (*n* = 11,551), (b) probes containing single-nucleotide polymorphisms within five base pairs of the targeted CpG site (*n* = 7,998), and (c) probes that cross-react with more than one region in the human reference genome (hg19) (*n* = 3,965). The publicly available data for training the MNP classifier were analyzed by using Illumina Infinium (H450k), while the in-house samples were analyzed using Illumina Infinium Human MethylationEPIC BeadChip (EPIC v1 and EPIC v2). Therefore, to be able to merge all arrays, common probes were used. A total of 358,403 probes were used in downstream analysis. For the unsupervised clustering, the same methods as previously described are used (1). The most variable 32,000 CpGs from all samples (publicly available 2,801 samples and 75 in-house samples) were filtered. Eigenvalue decomposition was performed, and 94 nontrivial components were used for t-distributed stochastic neighbor embedding (t-SNE) analysis. RSpectra version 0.16.2 and Rtsne version 0.17 were used for the analysis.

### Statistical analysis

Statistical analyses were conducted using GraphPad Prism 10, including calculations for means, standard deviation, Student’s *t*-tests, one-way ANOVA, correlations, and scatter plots.

## Results

### Cohort characteristics

The study included 96 pediatric patients with a mean age of 6.9 years at diagnosis. The cohort comprised 54 male patients and 42 female patients. Among the 75 CNS tumor cases, the majority were gliomas (*n* = 32), followed by embryonal tumors (*n* = 22). Non-CNS tumors (*n* = 11) included germ cell tumors, neuroblastomas, and soft tissue sarcomas, while non-tumoral CNS lesions (*n* = 10) included vascular malformations and epilepsy-associated lesions. Detailed demographic and clinical characteristics are summarized in **Table 1**.

### Assay performance of classifiers on the CNS tumors

To evaluate the performance of each classifier, we first focused on the CNS tumors and retained only one assay per patient.

**Figure 1:**
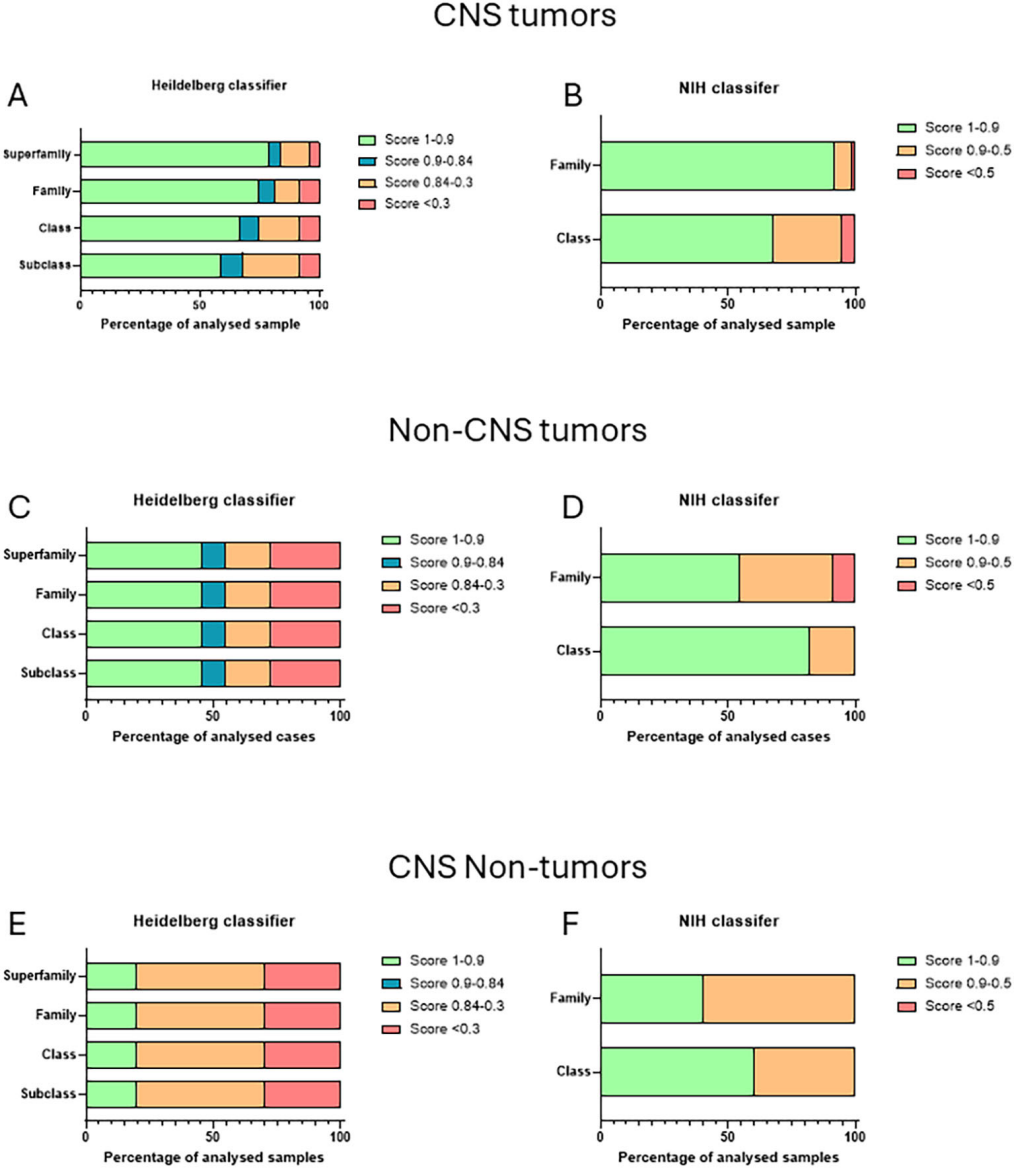
Classifier confidence across specimen categories. Percentage of the cases with score >0.9 (in green), between 0.9 and 0.84 (in blue), between 0.84 and 0.3 (in orange), and <0.3 (in red) at each level of classification with the Heidelberg classifier. For the CNS pediatric tumors **(A)**, non-CNS tumors, **(C)** and CNS non-cancer lesions **(E)**. Percentage of the cases with score >0.9 (in green), between 0.9 and 0.5 (in orange), and <0.5 (in red) at each level of classification with the NIH methylscape classifier. For the CNS pediatric tumors **(B)**, non-CNS tumors **(D)**, and CNS non-cancer lesions **(F)**.

For the Heidelberg classifier, using a calibrated score cutoff of ≥0.9 (as recommended by Capper et al. (1)), the Heidelberg classifier matched a methylation class at the superfamily level in 79% of patients (59/75) and at a subclass level in 59% of patients (44/75). With a lower cutoff of ≥0.84 (as suggested by Capper et al. (21)), this percentage increased to 84% (63/75) at the superfamily level and 68% (51/75) at the subclass level (**Figure 1A**).

With the NIH methylscape classifier, 92% (69/75) matched a methylation class at the superfamily level and 68% of patients (51/75) matched at the level with a score ≥0.9 (**Figure 1B**).

While we observed a very high concordance between the classification outputs of the two tools on individual CNS tumors, the distribution of calibrated scores differed slightly. At a threshold of 0.9, the NIH classifier yielded a modestly higher proportion of high confidence calls than the Heidelberg classifier, particularly at the subclass level (**Supplementary Figures S1A, B**). Based on the obtained scores by comparing the NIH methylscape classifier to the Heidelberg classifier, the scores obtained at the superfamily and class levels were significantly higher using the NIH classifier (**Supplementary Figure S1C**). It therefore appears that the level of confidence provided by the NIH methylscape classifier is slightly higher than the one provided by the Heidelberg classifier.

Finally, we calculated the F1 score for both classifiers, each yielding an F1 score of 0.936 based on 66 true positives, four false positives, and five false negatives. This result reflects the excellent overall performance and robustness of both classification models.

### Assay performance of the classifiers on non-CNS tumors and non-tumoral CNS lesions

Additionally, we investigated the possibility of analysis of non-CNS tumors and “CNS non-tumoral lesions” samples. Yet, to be relevant, the non-CNS tumors analyzed here (germ cell tumors, neuroblastomas, and sarcomas) are all tumor types included in the classifier’s reference database (detailed in **Table 1**).

The score obtained with non-CNS tumor were relatively high with 45.5% (5/11) with score >0.9 and 54.5% (6/11) with score >0.84 at the superfamily and subclass level with the Heidelberg classifier (**Figure 1C**). The scores were even higher with 54.5% (6/11) at the superfamily level and even 81.8% (9/11) at the subclass level using the NIH methylscape classifier (**Figure 1D**).

On the other hand, the scores obtained on the “CNS non-tumoral lesions” were comparatively low with 20% (2/10) with score >0.9 at the superfamily and subclass level with the Heidelberg classifier (**Figure 1E**).

Yet, all the ones with score >0.3 confirmed the “normal” condition of the tissue. On the same samples, 40% (4/10) at the superfamily level and even 60% (6/10) at the class level using the NIH methylscape classifier scored >0.9 (**Figure 1F**). Here three out of four of the high scores pointed to a tumor diagnosis, while histology revealed no such conditions. One cavernous angioma, one tuberous sclerosis, and one focal dysplasia type were all classified as low-grade glioma. The re-evaluation of the H&E confirmed that those were misclassifications from the NIH methylscape classifier, which, if taken at face value, could have inappropriately reclassified benign lesions as infiltrative glioma and prompted unnecessary oncologic workup or treatment in children.

### Evaluation of the impact of technical parameters on assay performance

**Figure 2:**
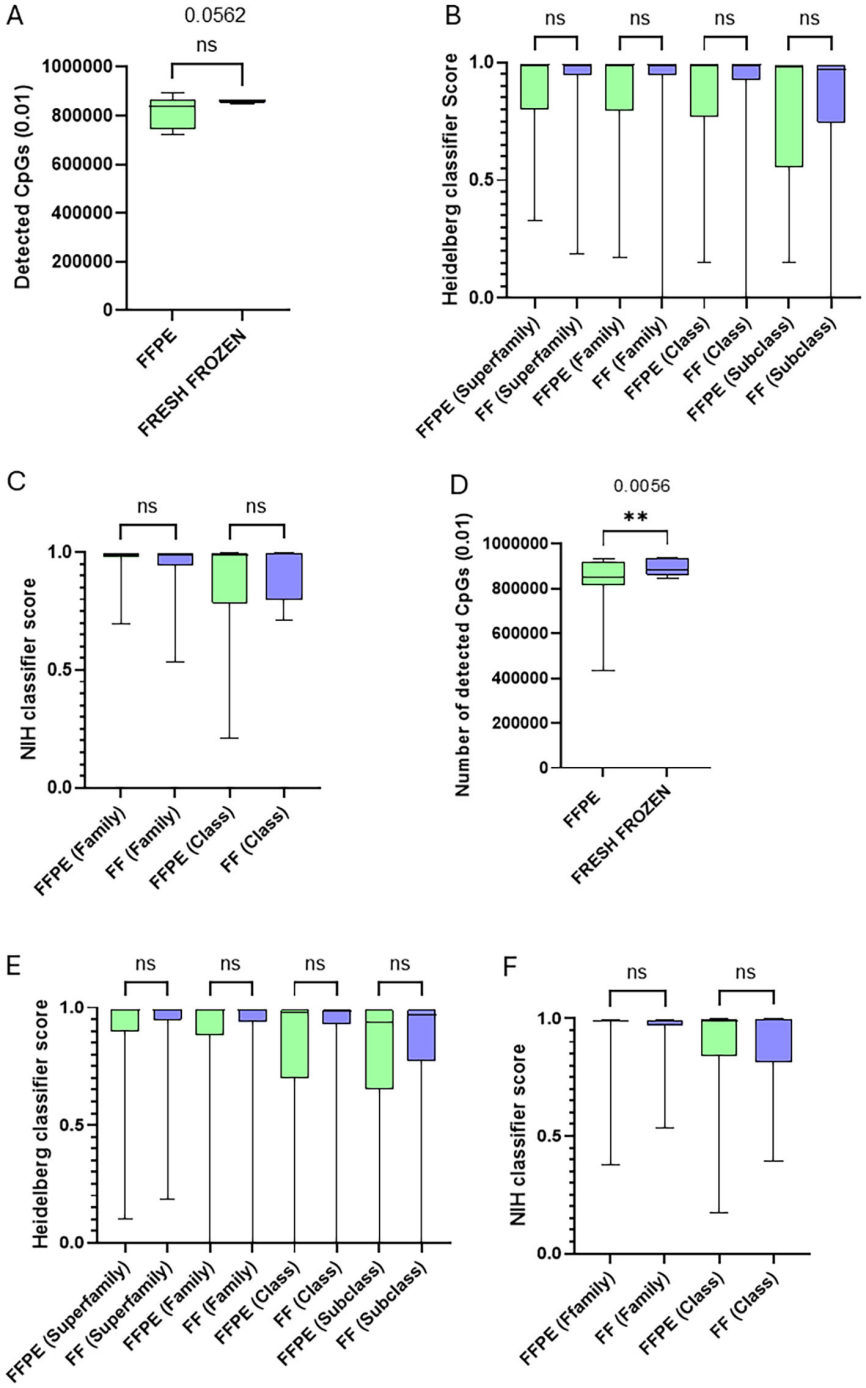
Tissue preservation has minimal impact on classifier scores. **(A)** Comparison of detected CpGs between paired FFPE and FF sample for the same patients revealed no statistical difference (paired *t*-test) (*p* = 0.0562). **(B)** Comparison of scores obtained for the nine pairs of FF and FFPE samples from the same patients at every level of classification of the Heidelberg classifier. The statistical comparison was performed using ANOVA. **(C)** Comparison of scores obtained for the nine pairs of FF and FFPE samples from the same patients at every level of classification of the NIH methylscape classifier. (The statistical comparison was performed using ANOVA.) **(D)** Comparison of detected CpGs between all unique FFPE (62 samples) and unique FF (26 samples) CNS tumor samples processed (unpaired *t*-test) (*p* = 0.0056). **(E)** Comparison of scores obtained for all the unique FFPE (62 samples) and unique FF (26 samples) CNS tumor samples processed at every level of classification of the Heidelberg classifier. The statistical comparison was performed using ANOVA. **(F)** Comparison of scores obtained for all the unique FFPE (62 samples) and unique FF (26 samples) CNS tumor samples processed at every level of classification of the NIH methylscape classifier. The statistical comparison was performed using ANOVA. **p<0.01.

Both classifiers can be used with DNA isolated from FFPE and FF samples. To investigate whether the use of fresh frozen samples could enhance diagnostic performance by the classifiers, we included nine pairs of matching fresh frozen and FFPE CNS tumor samples. The number of CpGs detected was trending higher in FF samples (*p* = 0.0562) compared to FFPE samples (**Figure 2A**). However, no statistical difference in the Heidelberg classifier scores (**Figure 2B**) nor in the NIH methylscape classifier scores (**Figure 2C**) was observed at any level of classification (superfamily to subclass). Similarly, when comparing all different CNS tumor FFPE samples analyzed in our cohort (62 samples) with all CNS tumor FF samples (26 samples), we once more observed a higher average number of CpGs detected (*p* = 0.0056) in FF samples (**Figure 2D**), but there was once more no statistical difference in both classifiers’ scores between the two groups at any classification level (**Figures 2E, F**).

Finally, a T-SNE analysis of the samples based on FFPE *vs*. FF segregation was performed and showed no clustering effect based on whether the samples were FFPE or FF, reinforcing the fact that both can be used for analysis and to highlight the robustness of methylation profiling across different sample types (**Supplementary Figure S2**).

We also explored whether the number of analyzable CpGs was correlated with the score obtained. We identified a weak positive correlation between the analyzed CpGs number and the Heidelberg classifier scores at some classification level (*p* = 0.0082, *R*^2^ = 0.078 for scores at the superfamily level; *p* = 0.54, *R*^2^ = 0.0042 for scores at the family level; *p* = 0.022, *R*^2^ = 0.060 for scores at the class level; and *p* = 0.010, *R*^2^ = 0.074 for scores at the subclass level, respectively) (**Supplementary Figures S3A–D**).

Similarly, with the NIH methylscape classifier, the correlation between the analyzed CpG number and the Heidelberg classifier scores was not significant at the superfamily level (*p* = 0.90, *R*^2^ = 0.00018), but it was at the class level (*p* = 0.018, *R*^2^ = 0.063) (**Supplementary Figures S3E, F**).

**Figure 3:**
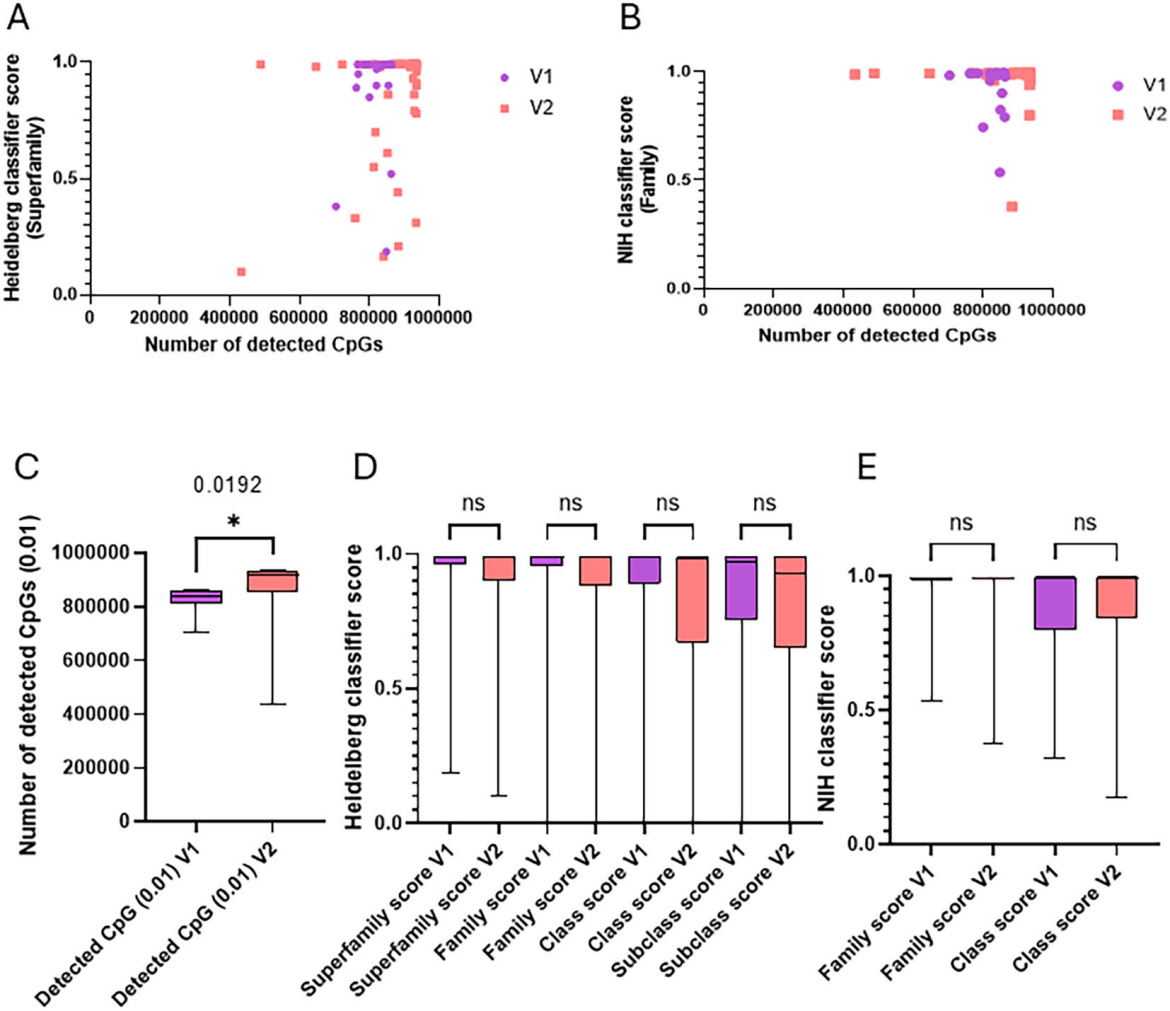
EPIC v2 increases CpG detection without altering classifier scores. **(A)** Dot plot of the number of detected CpGs with corresponding Heidelberg score at the superfamily level. In violet are the samples processed using Illumina MethylationEPIC v1.0 Kit (20 FFPE and 13 FF samples), and in red are the samples processed using Illumina MethylationEPIC v2.0 Kit (42 FFPE and 13 FF samples). **(B)** Dot plot of the number of detected CpGs with corresponding NIH methylscape score at the superfamily level. In violet are the samples processed using Illumina MethylationEPIC v1.0 Kit, and in red are the samples processed using Illumina MethylationEPIC v2.0 Kit. **(C)** Comparison of the numbers of detectable CpGs on samples using V1 or V2 Illumina MethylationEPIC kits. Significantly more CpGs were detected with V2 kits (*p* = 0.0192, unpaired *t*-test). **(D)** Comparison of scores obtained for all of the samples processed with Illumina MethylationEPIC v1 (33 cases) and v2 (55 cases) CNS tumor samples at every level of classification of the Heidelberg classifier. The statistical comparison was performed using ANOVA. **(E)** Comparison of scores obtained for all of the samples processed with Illumina MethylationEPIC v1 (33 cases) and v2 (55 cases) CNS tumor samples at every level of classification of the NIH methylscape classifier. The statistical comparison was performed using ANOVA. * p < 0.05.

Despite these correlations, the plots of CpGs with scores show that a higher number of analyzable CpGs does not guarantee a higher score with neither of the classifiers, even at the superfamily level of classification (**Figures 3A, B**).

Since we used both the Illumina 850k BeadChip (v1) in 2023 and the 930k BeadChip (v2) in 2024, we distinguished between the two bead chip types. We found that, as expected, more analyzable CpGs were recovered with the v2 chip than with v1 (*p* = 0.019) (**Figure 3C**). However, since the CpGs used for classification were defined among the 850k CpGs present in v1 (and still present in v2), the additional CpGs recovered with v2 did not improve the average scoring with either classifier at any level of classification (**Figures 3D, E**).

Finally, despite efforts to optimize DNA extraction and using the recommended 500 ng by Illumina for EPIC analysis, we were unable to consistently recover the required amount of DNA. In some cases, we performed analysis with as little as 300 ng, which became our internal cutoff (above the 250 ng recommended by Illumina). Interestingly, using the recommended DNA amount did not guarantee a high number of CpGs recovered or a good score. Conversely, using low DNA quantities (as low as 300 ng) could still result in a good number of CpGs detected and a DKFZ score ≥0.9 (**Supplementary Figures S4A–E**).

### Assay reproducibility

We included one particular sample (a medulloblastoma) at each Illumina MethylationEPIC experiment as an internal control across the 17 separate experiments that we ran. While the scores obtained at the superfamily and family levels were nearly identical across all 17 experiments, some score discrepancies were seen at the class and subclass level with the Heidelberg classifier (**Supplementary Figure S5A**). This minor variation observed in the Heidelberg score was not seen with the NIH methylscape classifier (**Supplementary Figure S5B**). These minor variations were not linked with the freeze–thaw cycles of the sample nor was the number of CpGs detectable (**Supplementary Figure S5C**). Similarly, two additional samples were respectively run three times and had very similar results at each run with both classifiers (**Supplementary Figures S5D–I**).

### Primary tumor and relapse comparison

One patient with atypical teratoid/rhabdoid tumor underwent three surgeries for a primary tumor and two relapses. All three analysis (FFPE samples) gave a similar diagnostic from the classifiers with scores ≥0.84 and >0.9, respectively (**Supplementary Figures S6A, B**). Interestingly, the diagnostic of the primary tumor had a score slightly lower than the relapse, but that might be only due to the purity of the cancer tissue present within the selected block. Similarly, two other patients with ependymomas (both frozen samples) and adamantinomatous craniopharyngioma (both FFPE samples) had primary and relapse analyzed. Like the previous example, both primary and relapse returned the same classification with similar scores with the Heidelberg classifier (**Supplementary Figures S6C–F**). For the ependymomas, a lower score (<0.5) at the class level with the NIH classifier requalified the subclass 1E as subclass 1D, leading to a minor discrepancy between the classifiers.

### Diagnostic performance of the classifiers

We observed a very high concordance in the classification between the two classifiers. Indeed, within the CNS tumors, the two classifiers match together at the superfamily level in 98.66% (74/75) of cases and in 86.66% (65/75) of cases at the class level (**Supplementary Figures S1A, B**). The discrepancy observed at the class level were often very minor, such as posterior fossa group A (pfa) ependymoma, subclass 1b for the Heidelberg classifier versus posterior fossa group A (pfa) ependymoma, subclass 1c for the NIH classifier. The rest of the discordance at the class level were due to very low score obtained in either or both classifiers. The scores obtained with the NIH classifier were slightly higher at the superfamily level (*p* = 0.0008) and class level (*p* = 0.008) between the two classifiers (**Supplementary Figure S6C**).

**Figure 4:**
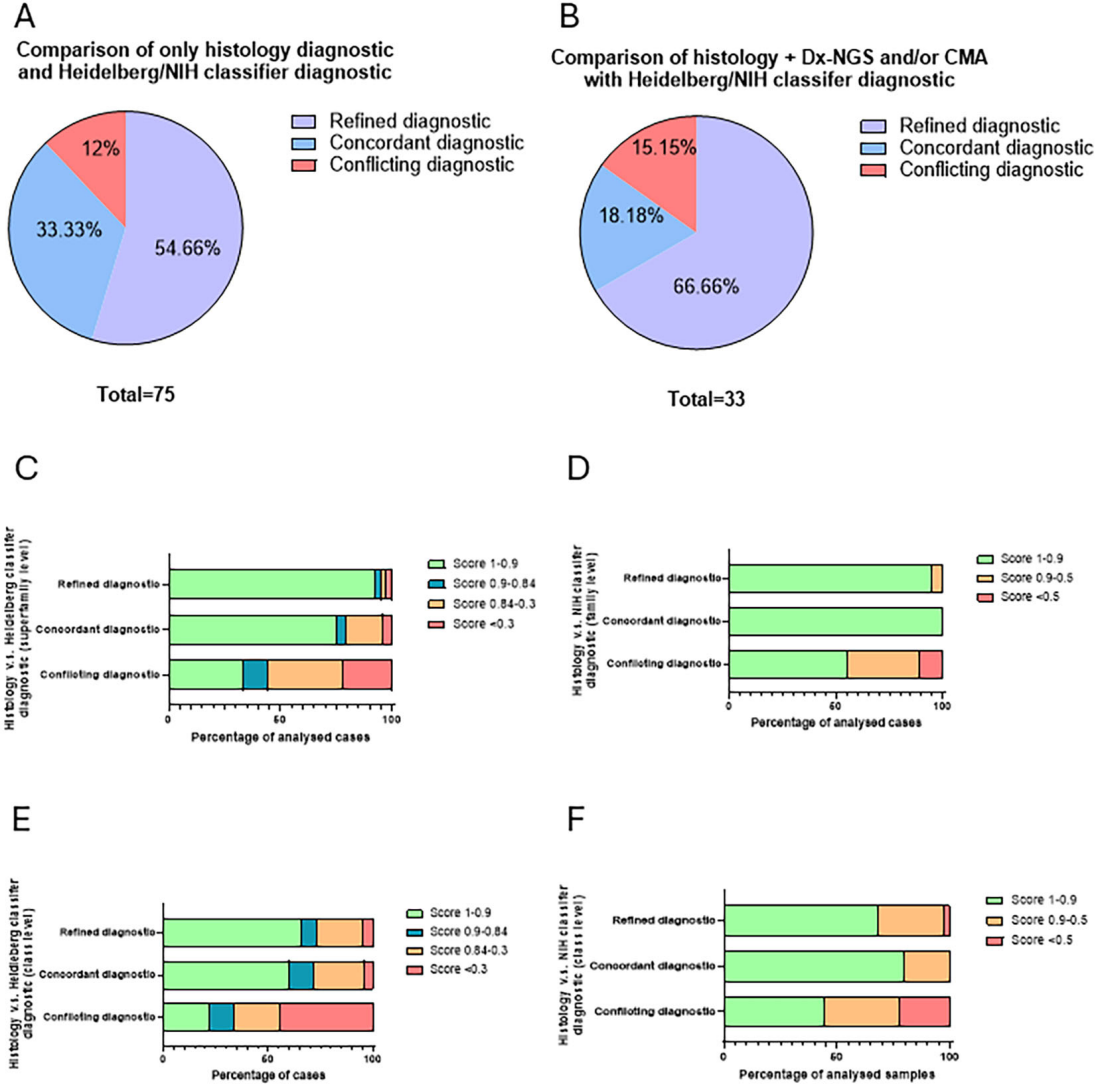
Diagnostic impact versus integrated histopathology. **(A)** Pie chart of the percentage of the 75 CNS tumors with refined (purple), concordant (blue), and conflicting (red) diagnostic provided by the classifiers compared to the histology diagnostic alone. The numbers are identical with both classifiers; therefore, the same pie chart is representative for both classifiers. **(B)** Pie chart of the percentage of the 33 CNS tumors with refined (purple), concordant (blue), and conflicting (red) diagnostic provided by the classifiers compared to the histology diagnostic complemented with Dx-NGS and CMA data. **(C)** Percentage of the 75 CNS tumor cases with score >0.9 (in green), between 0.9 and 0.84 (in blue), between 0.84 and 0.3 (in orange), and <0.3 (in red) for each concordance between histology only diagnostic and Heidelberg classification (refined, concordant, and conflicting) at the superfamily level **(C)** and the subclass level **(E)**. **(D)** Percentage of the 75 CNS tumor cases with score >0.9 (in green), between 0.9 and 0.5 (in orange), and <0.5 (in red) for each concordance between histology only diagnostic and NIH methylscape classification (refined, concordant, and conflicting) at the superfamily level **(D)** and the subclass level **(F)**.

We then assess the correspondence between histology diagnostic and the classifiers’ prediction for each of the 75 patients. We observed that in 88% (66/75) of cases the classifier confirmed the diagnostic posed by the initial pathological diagnosis (based on histology alone), and in 54.7% (41/75) the classifier provided a more refined diagnosis than the one identified with histology alone (**Figure 4A**). In only 12% (9/75), we obtained a conflicting diagnosis between histology and the classifiers.

Among the 75 cases, we obtained diagnostic next-generation sequencing (Dx-NGS) and/or chromosomal microarray analysis (CMA) for 33 patients. Among these patients, both classifiers provided matching diagnostic in 84.9% (28/33) of cases with the diagnostic obtained with histology and molecular analysis and the classifiers’ diagnostic. In 15.1% (5/33), we had discordant diagnostic (**Figure 4B**). Surprisingly, even with the integration of next-generation sequencing (NGS), the proportion of cases in which the classifier provided a more refined diagnosis was higher than the global average (66.6% *vs*. 54.65%, respectively). This discrepancy may reflect the inclusion of more complex cases that necessitate advanced molecular characterization.

Besides that, the score obtained with Heidelberg and NIH classifier in the concordant and refined diagnostic, at the superfamily and subclass level, were usually high compared to the conflicting ones (**Figures 4C, E**). The conflicting diagnostics were most often due to low scores. Notably, molecular validation via NGS (Dx-NGS) and chromosomal microarray (CMA) yielded higher scores in refined diagnostics compared to the conflicting group; however, concordant cases maintained a marginal score advantage over the refined group (**Figures 4D, F**).

Among the 75 CNS tumor cases, nine (12%) exhibited conflicting diagnoses between histopathology and the methylation classifiers (**Figure 4A**). Four cases had low classifier scores (≤0.84 for Heidelberg and ≤0.5 for NIH), with two classified as normal tissue, likely due to low tumor content in the analyzed FFPE blocks despite representing >70% of the tissue on H&E slides. The remaining five cases had high classifier scores (≥0.84 for Heidelberg and ≥0.9 for NIH) but conflicted with histopathological diagnoses, which were confirmed upon H&E reinspection and additional molecular testing (Dx-NGS) (**Figure 4B**) and are therefore true conflicts.

### UMAP/t-SNE analysis

**Figure 5:**
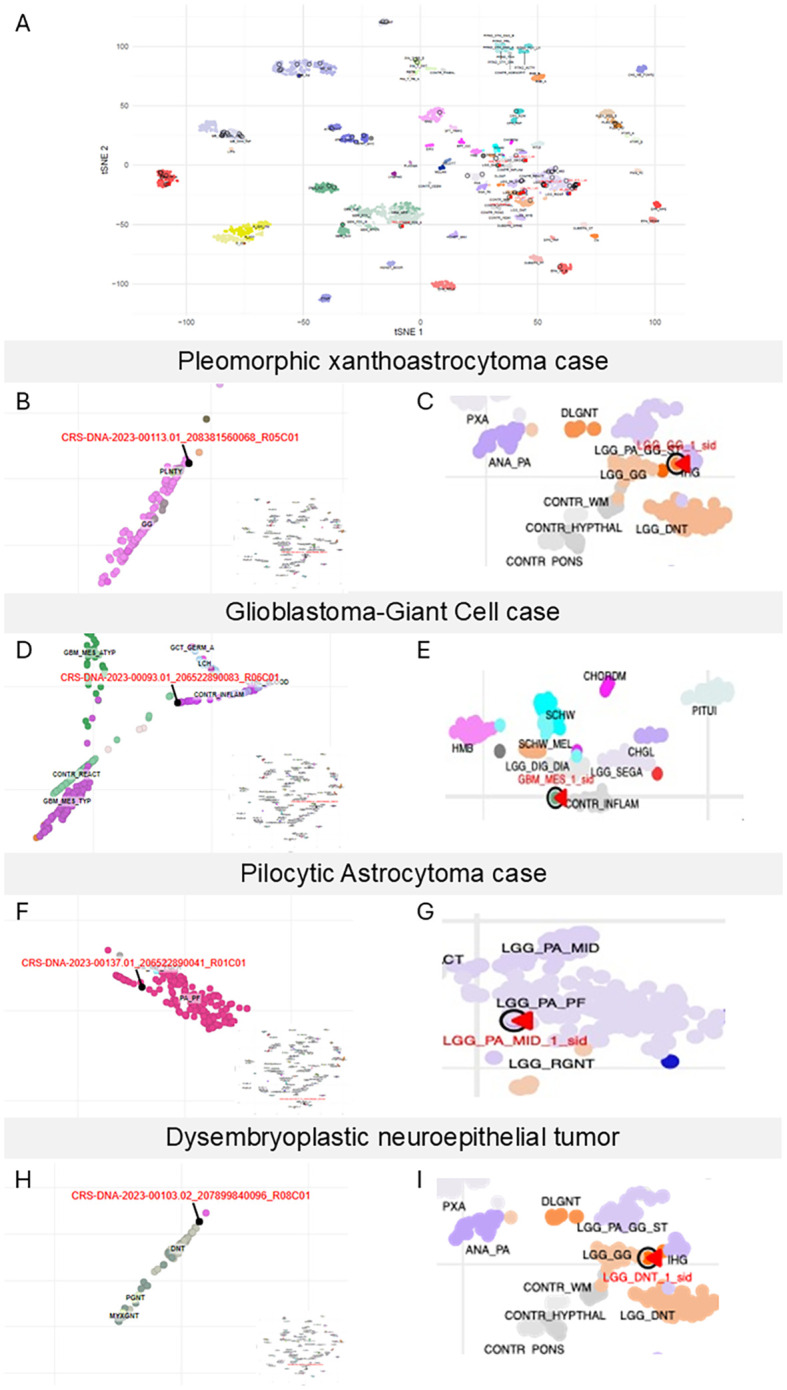
Case-level embeddings highlight concordant and discordant assignments. **(A)** t-SNE representation of all the 75 CNS tumors (circled in red) against the 2,801 samples published in Capper et al. Our samples are circled in black, and the samples not falling in their designated category (outliers) are indicated with a red arrow. Focused localization on the pleomorphic xanthoastrocytoma case on the NIH classifier of UMAP **(B)** and focus around this case on the t-SNE performed in our laboratory (labeled in red as LGG_GG_1_sid on the graph) **(C)**. Polymorphous low-grade neuroepithelial tumor (PLNTY) and ganglioglioma **(GG)** in the NIH classifier **(B)**. Low-grade glioma, ganglioglioma (LGG_GG), infant high-grade gliomas (IHG), in the t-SNE **(C)**. Focused localization of the glioblastoma–giant cell case on the NIH classifier of UMAP **(D)** and focus around this case on the t-SNE performed in our laboratory (labeled in red as GBM_MES_1_sid on the graph) **(E)**. Control inflammatory (CONTR_INFLAM), Langerhans cell histiocytosis (LCH), glioblastoma, IDH-wt, mesenchymal subtype, atypical (GBM_MES_ATYP) in the NIH classifier **(F)**. Subependymal giant cell astrocytoma (LGG_SEGA), control inflammatory (CONTR_INFLAM), desmoplastic infantile ganglioglioma (LGG_DIG), Schwannoma (SCHW_MEL) in the t-SNE (**E**). Focused localization on the pilocytic astrocytoma case on the NIH classifier of UMAP **(F)** and focus around this case on the t-SNE performed in our laboratory (labeled in red as LGG_PA_MID_1_sid on the graph) (**G**). Pilocytic astrocytoma, posterior fossa (PA_PF), infratentorial pilocytic astrocytoma subclass FGFR1-altered (PA_INF_FGFR) in the NIH classifier. Pilocytic astrocytoma, posterior fossa subclass (LGG_PA_PF), MC rosette-forming glioneuronal tumor (LGG_RGNT) in the t-SNE (**G**). Focused localization on the dysembryoplastic neuroepithelial tumor case on the NIH classifier of UMAP **(H)** and focus around this case on the t-SNE performed in our laboratory (labeled in red as LGG_DNT_1_sid in the graph) **(I)**. Dysembryoplastic neuroepithelial tumor (DNT), papillary glioneuronal tumors (PGNT) in the NIH classifier **(H)**. Low-grade glioma, ganglioglioma, (LGG_GG), infant high-grade gliomas (IHG), control tissue (CONTR_WM), dysembryoplastic neuroepithelial tumor (LGG_DNT) in the t-SNE **(I)**.

We utilized the online database and script published by Capper et al. to generate t-SNE plots and also employed the UMAP provided by the NIH classifier for clustering analysis of our methylation data. **Figure 5A** presents the t-SNE plot for our 75 CNS tumor samples mapped against 2,801 samples used to create the Capper et al. classifier in their first publication (1). Most samples were clustered within the expected subtype group; however, several outliers were observed, indicating discrepancies between histology and classification results. Most, but not all, outliers were of low scores, and the analysis of the UMAP/t-SNE could shed light onto the classification of those samples and their scores—for example, one such outlier was a pleomorphic xanthoastrocytoma (PXA) sample, classified as low-grade ganglioglioma by both classifiers despite histological diagnosis of PXA. In the NIH UMAP (**Figure 5B**), this sample clustered with the ganglioglioma group, explaining its high NIH classifier score. However, in the t-SNE plot (**Figure 5C**), it clustered between infantile high-grade glioma (IHG) and low-grade glioma ganglioglioma (LGG-GG), showing a Heidelberg classifier score of 0.86 at the superfamily level and 0.77 at the subclass level.

Another outlier, a glioblastoma-giant cell, showed high scores at the superfamily and class levels but lower subclass scores. The NIH UMAP clustered this sample with control inflammation (**Figure 5D**), suggesting potential issues with sample purity or contamination. The t-SNE plot for the same sample (**Figure 5E**) further separated this sample from any distinct cluster.

A pilocytic astrocytoma (PA) demonstrated a discrepancy between the Heidelberg and NIH classifiers (at the subclass level), with the Heidelberg classifying it as PA, midline. The NIH classifier provided a high-confidence score than the Heidelberg one. Both NIH UMAP and our t-SNE clustered this sample within the PA_PF (pilocytic astrocytoma, posterior fosa) group (**Figures 5F, G**).

A rare DNT sample was correctly identified by both classifiers despite low scores. The NIH UMAP but not t-SNE clustering confirmed the diagnosis (**Figures 5H, I**), increasing confidence in the result for the NIH classifier.

## Discussion

In pediatric CNS tumors, methylation classification agreed with integrated histopathology in 88.0% (66/75) and refined diagnoses in 54.7% (41/75), quantifying its added value beyond routine morphology. These results are consistent with prior pediatric series (≈49%–95% concordance) (1, 8, 9, 26). It frequently refined diagnoses (54.7%), providing clinically actionable specificity. Applied with clearly defined, locally calibrated score thresholds and pathology-first adjudication, methylation classifiers function as decision-support tools in pediatric CNS workflows, consistent with WHO 2021 guidance (27, 28) provided that their output is interpreted within joint pathological and multidisciplinary review rather than as fixed, stand-alone rules.

Classifier scores were comparable across FF and FFPE and unchanged by the EPIC v1→v2 update, consistent with prior reports (8, 23). FF did not confer a scoring advantage over FFPE under our conditions. DNA input alone did not predict performance: low-yield samples sometimes reached sufficient CpG coverage and accurate calls, whereas a high input did not guarantee optimal scores. EPIC v2 increased CpG detection as expected without materially shifting scores, supporting the use of either version.

The remaining failure modes concentrated in non-neoplastic CNS lesions and a small subset of atypical tumors. Non-neoplastic CNS lesions yielded lower scores, and NIH methylscape occasionally produced confident tumor labels that required pathologist adjudication. In our series, cavernous angioma, tuberous sclerosis and focal cortical dysplasia samples were all assigned low-grade glioma classes, illustrating how overreliance on classifier output could, in principle, trigger an erroneous diagnosis of glioma, additional imaging or surgery, and consideration of adjuvant therapy or intensified surveillance for patients who do not harbor a neoplasm. Our workflow mitigated this risk because every output was interpreted in the context of histology, radiology, and clinical information, and the benign nature of these lesions was ultimately retained. These observations emphasize that adequate tumor content and joint pathological review are essential for safe clinical integration and that methylation classifiers should remain complementary to, rather than replacing, conventional diagnostic pathways. To contextualize borderline or discordant outputs, we generated UMAP/t-SNE embeddings from the 32,000 most variable CpGs in our cohort, whereas the Heidelberg model operate on ~10,000 training-selected CpGs. This broader CpG context provides additional genome-wide signal and often places low-confidence cases within biologically coherent neighborhoods on the CNS methylation landscape—clarifying whether a score is directionally plausible versus spurious. (Embeddings were unsupervised and not used to make the primary call).

Embedding projections (UMAP/t-SNE) plus orthogonal tests (IHC, Dx-NGS, and CMA) resolved ambiguity in complex or rare tumors, including the four high-confidence conflicts. Our representative cases (PXA, GBM-GC, PA, and DNT) illustrate technical and biological drivers of discordance in morphologically heterogeneous tumors and show how embedding-space context can guide adjudication.

Reproducibility is critical for clinical integration. In our study, the use of inter-experiment controls—specifically, a designated control sample—helped identify a technical issue in one batch of analyses, where the control sample showed significantly lower CpG detection and failed to reach a score ≥0.9. This underscores the importance of internal controls and the inclusion of technical parameters, such as tumor purity estimates, DNA quantity, bisulfite conversion quality, and detected CpG sites, in diagnostic reports to facilitate an accurate interpretation of results. Because the underlying machine learning models operate on these quantitative inputs, variability in tumor purity and DNA quality can shift calibrated scores, so any numerical thresholds should be applied with flexibility and interpreted alongside morphological and technical context. Additionally, the batching requirement for processing samples in groups of eight caused delays in diagnostic turnaround time, prompting exploration of alternative methods like Oxford Nanopore Technology sequencing, which could improve workflow efficiency.

High-confidence outputs (≥0.90) were interpreted within neuropathology; any discordance triggered targeted IHC/Dx-NGS/CMA. Running two classifiers in parallel increased confidence when concordant; discrepant or low-confidence outputs served as diagnostic leads that required MDT adjudication integrating histology, IHC, molecular findings, and clinical context. Classifier disagreement should prompt additional scrutiny—not automatic deferral to one tool—highlighting the importance of human expertise in interpreting computational outputs. Our experience suggests that clear, pre-agreed score thresholds combined with joint pathological review are particularly important in resolving the rare but high-confidence conflicts where algorithmic certainty and integrated histology diverge. Even very high classifier scores should therefore not bypass multidisciplinary interpretation, especially in lower-purity or technically suboptimal samples.

In 5.3% (4/75), high-confidence classifier labels were overturned on integrated review—slightly lower than the 6%–25% range reported by Capper et al. (18). In all four cases, original histological diagnoses were retained after comprehensive re-evaluation, including H&E review and integration of molecular data. These cases underscore the critical role of methylation profiling as a diagnostic aid that can raise flags and refine subtyping but should never be used in isolation. They also highlight the need for interpretive caution when classifier outputs conflict or when biological complexity reduces classification certainty. As illustrated by several cases in our study (e.g., PXA, GBM-GC, and DNT), resolving diagnostic uncertainty requires synthesizing all available data—morphological, molecular, and computational—and maintaining a central role for expert pathological assessment. Operationally, pre-specified thresholds and reflex orthogonal testing keep the pathologist central and the process reproducible.

Several limitations warrant further investigation. First, this is a single-center study from a tertiary pediatric institution, which may limit generalizability to other populations, health systems, and practice settings. Second, although the Heidelberg and NIH/Bethesda classifiers were trained on thousands of reference samples, our evaluation cohort is comparatively small, particularly for rare tumor subtypes and recurrent tumors, which reduces power to detect subgroup-specific failure modes. Third, the retrospective design introduces potential selection bias and incomplete metadata capture, both of which may influence estimates of classifier accuracy and reproducibility. We did not systematically quantify tumor purity or model the impact of DNA quality and CpG detection on calibrated classifier scores, so the reliability of classifier-based diagnoses and the generalizability of our proposed thresholds may be lower in settings with different pre-analytic conditions. This further underscores that classifier output should be interpreted within interdisciplinary review, particularly in diagnostically challenging or rare entities. Furthermore, we did not perform a formal analysis of cost or turnaround time, even though these operational parameters are critical for workflow integration and real-world implementation. Additionally, the integration of methylation profiling results into multidisciplinary team discussions, as recommended by the Haarlem and ICCR guidelines, is essential for comprehensive diagnostic evaluations. Routine integration into multidisciplinary tumor boards (Haarlem/ICCR) and public release of data and analysis code will aid replication; multi-site pediatric cohorts should assess sample-age effects, prospective clinical utility, and health-economic impact. Ultimately, methylation profiling should serve as an adjunct to traditional methods, guiding clinicians in challenging cases and aiding in the identification of new tumor subtypes.

## Data Availability

The original contributions presented in the study are included in the article/**Supplementary Material**, further inquiries can be directed to the corresponding author/s.

## Supplementary material

Supplementary Figure 1 Head-to-head Heidelberg versus NIH performance. Pie chart of the percentage of the 75 CNS tumors concordance of classification between Heidelberg and NIH methylscape classifiers at the superfamily (Heidelberg)/family (NIH) level **(A)** and at the class level **(B)**. **(C)** Comparison of the scores obtained at the superfamily level and class level with both Heidelberg and NIH classifiers. Significantly higher scores were obtained with NIH classifier at the superfamily (Heidelberg)/family (NIH) (*P* = 0.0008, paired *t*-test) and class level (*P* = 0.008).

Supplementary Figure 2 No material-driven clustering on UMAP. Uniform manifold approximation and projection (UMAP) visualization of DNA methylation profiles from FFPE and FF samples. The plot demonstrates no significant clustering based on sample type (FFPE *vs*. FF).

Supplementary Figure 3 Relationship between detectable CpGs and classifier scores. Dotplot of 88 samples (duplicates excluded) with detectable CpGs against the Heidelberg score at the superfamily **(A)**, family **(B)**, class **(C)**, and subclass level **(D)**. Linear regression with *R*^2^ (up) and *P*-value (down) are provided for each. Dotplot of 88 samples (duplicates excluded) with detectable CpGs against the NIH methylscape score at the family **(E)** and class level **(F)**. Linear regression and *P*-value and *R*^2^ are given for each.

Supplementary Figure 4 Array version effects visualized. **(A)** Dotplot of 88 samples (duplicates excluded) with detectable CpGs against the quantity of DNA used for analysis. Samples ran on V1 (33 samples) are presented in violet, and samples ran on V2 are presented in red. **(B)** Dotplot of 88 samples (duplicates excluded) with Heidelberg scores at the superfamily level against the quantity of DNA used for analysis. Samples ran on V1 (33 samples) are presented in violet, and samples ran on V2 are presented in red. **(C)** Dotplot of 88 samples (duplicates excluded) with NIH methylscape scores at the family level against the quantity of DNA used for analysis. Samples ran on V1 (33 samples) are presented in violet, and samples ran on V2 are presented in red.

Supplementary Figure 5 Technical reproducibility across repeated control runs **(A**, **D**, **G)**. Cumulative Heidelberg classifier scores (superfamily (green), family (cyan), class (blue), and subclass (purple)) obtained for the first control sample ran 17 times **(A)**, the second control sample ran three times **(D)**, and the third control sample ran three times **(G)** (medulloblastoma) (in order of run). **(B, E, H)** Cumulative NIH methylscape classifier scores (family (green) and class (blue)) obtained for the first control sample ran 17 times **(B)**, the second control sample ran three times **(E)**, and the third control sample ran three times **(H)** (medulloblastoma) (in order of run). **(C**, **F**, **I**) Number of CpGS detected for each of the runs of the same first control sample (medulloblastoma) (in order of run) **(C)**, the second control sample **(F)**, and the third control sample (**I**).

Supplementary Figure 6 Primary–relapse longitudinal comparison. **(A, C, E)** Cumulative Heidelberg classifier scores (superfamily (green), family (cyan), class (blue), and subclass (purple)) obtained for the teratoid/rhabdoid tumor for primary tumor, first relapse, and second relapse **(A)**, the ependymomas samples ran for primary tumor, first relapse **(B)**, and the adamantinomatous craniopharyngioma sample ran for primary tumor, first relapse. **(B**, **D**, **F)** Cumulative NIH methylscape classifier scores (family (green) and class (blue)) obtained for the teratoid/rhabdoid tumor for primary tumor, first relapse, and second relapse **(A)**, the ependymomas samples ran for primary tumor, first relapse **(B)**, and the adamantinomatous craniopharyngioma sample ran for primary tumor, first relapse.
