# Supplementary material for: Enhancing the accuracy of molecular classification of pediatric CNS tumors: a dual-classifier approach using DNA methylation profiling
Source: Front Oncol. 2026 Feb 5;15:1701113. doi: 10.3389/fonc.2025.1701113 (PMC12916412; doi:10.3389/fonc.2025.1701113)
Supplement: Supplementary Figure 1 — Head-to-head Heidelberg versus NIH performance. Pie chart of the percentage of the 75 CNS tumors concordance of classification between Heidelberg and NIH methylscape classifiers at the superfamily (Heidelberg)/family (NIH) level (A) and at the class level (B). (C) Comparison of the scores obtained at the superfamily level and class level with both Heidelberg and NIH classifiers. Significantly higher scores were obtained with NIH classifier at the superfamily (Heidelberg)/family (NIH) (P = 0.0008, paired t-test) and class level (P = 0.008). [file Presentation1.pptx]

## Slide 1
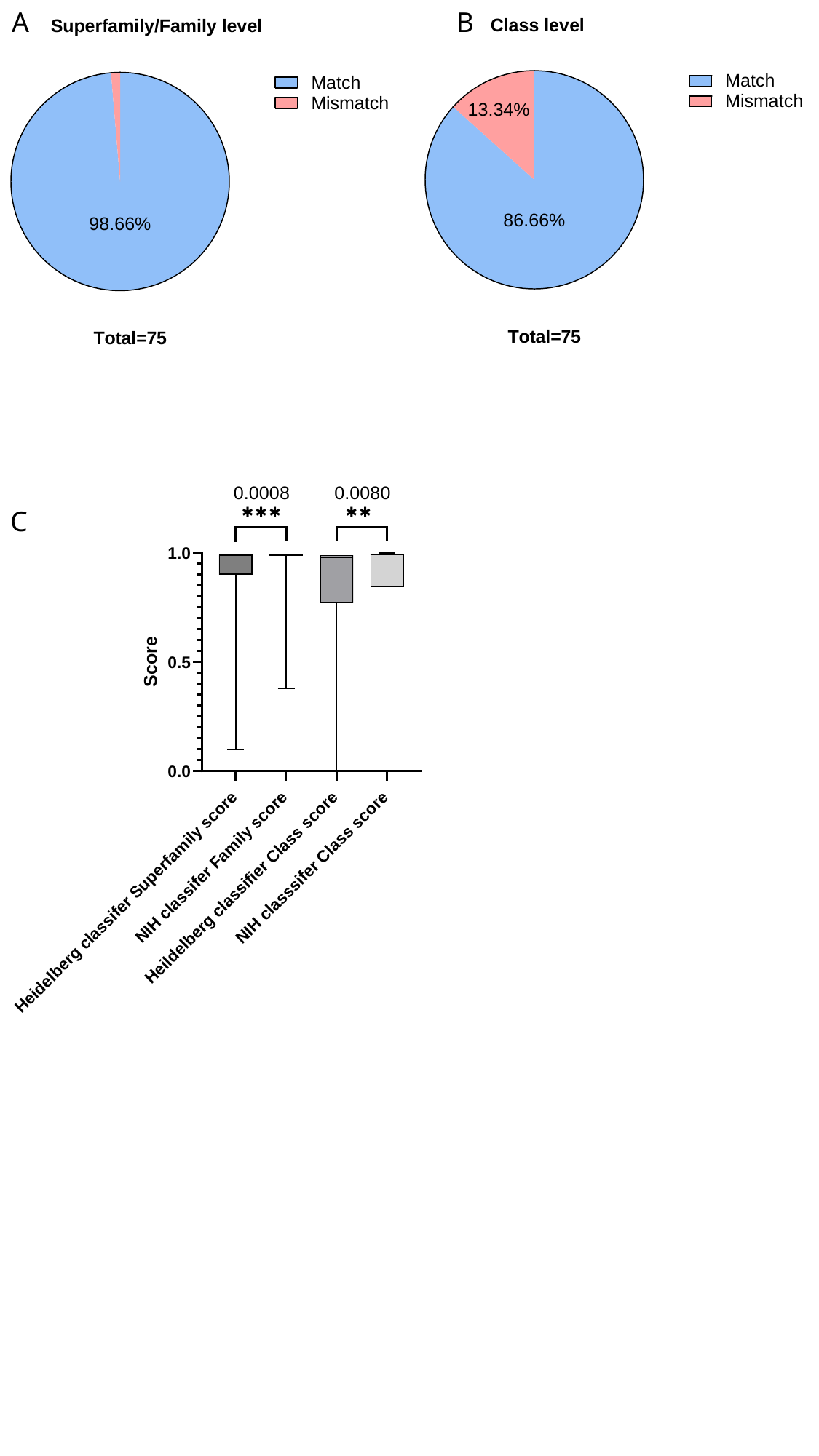

Supplementary Figure 1
A
B
C

## Slide 2
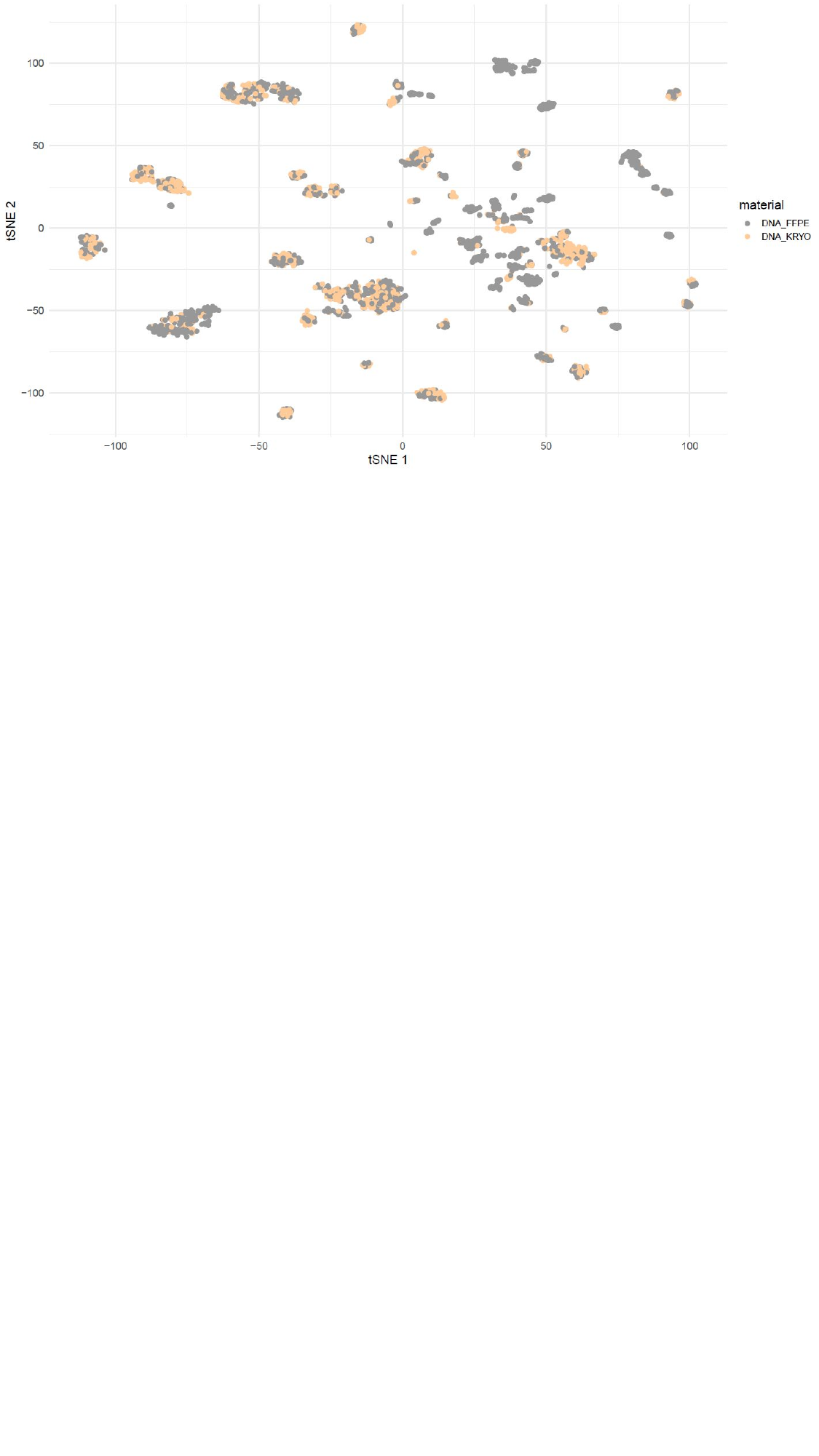

Supplementary Figure 2

## Slide 3
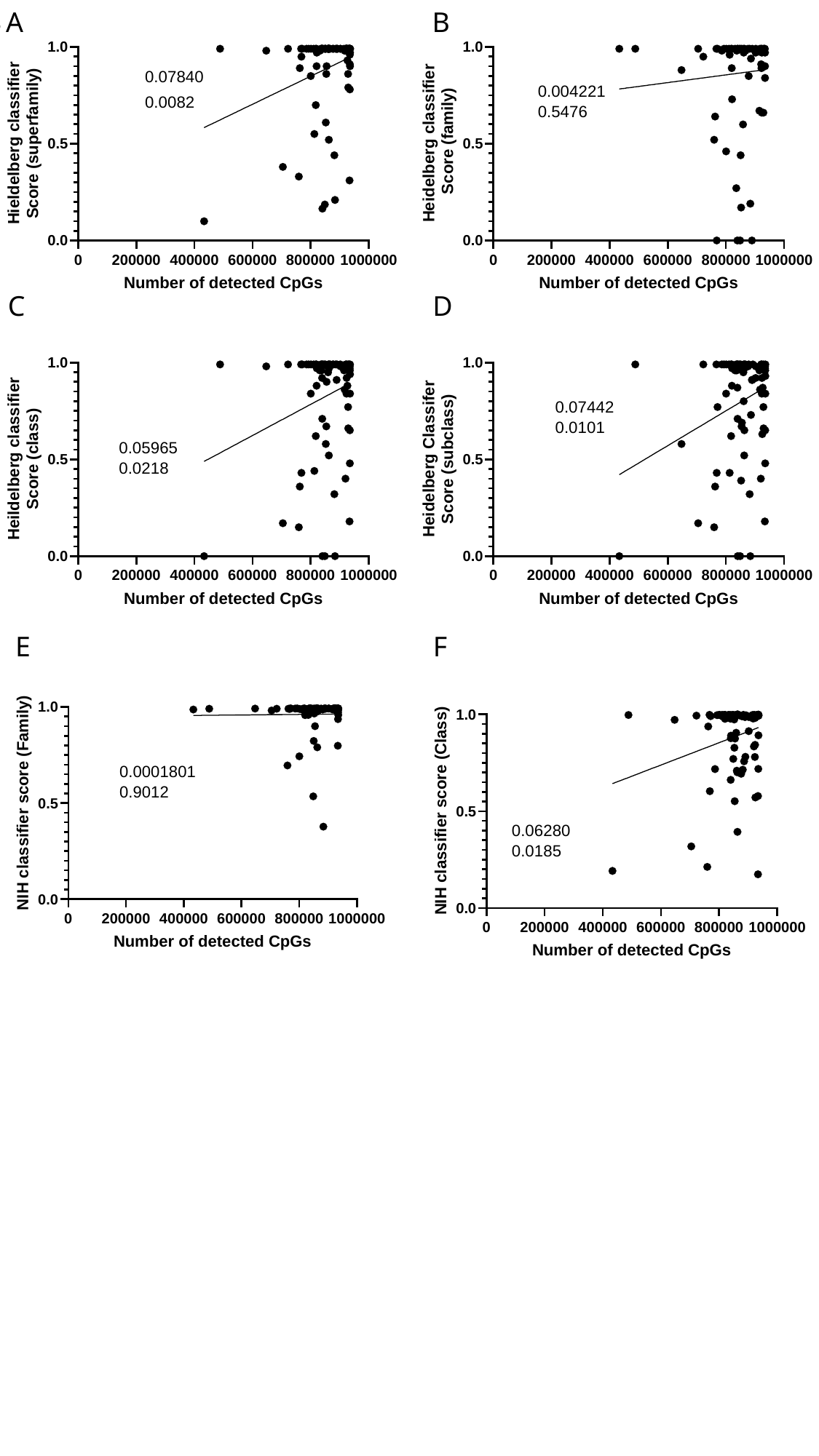

Supplementary Figure 3
A
B
C
D
E
F

## Slide 4
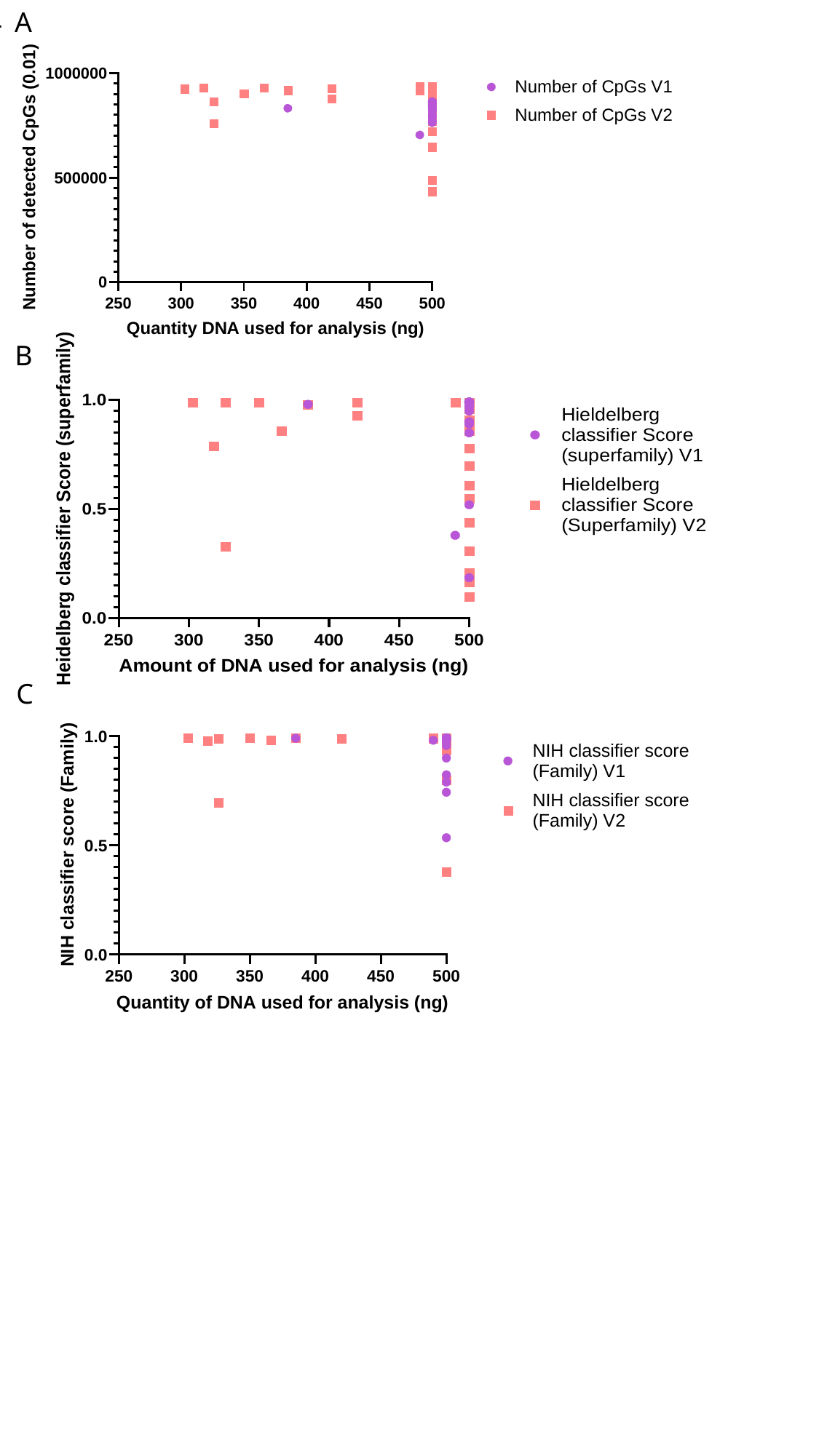

Supplementary Figure 4
A
B
C

## Slide 5
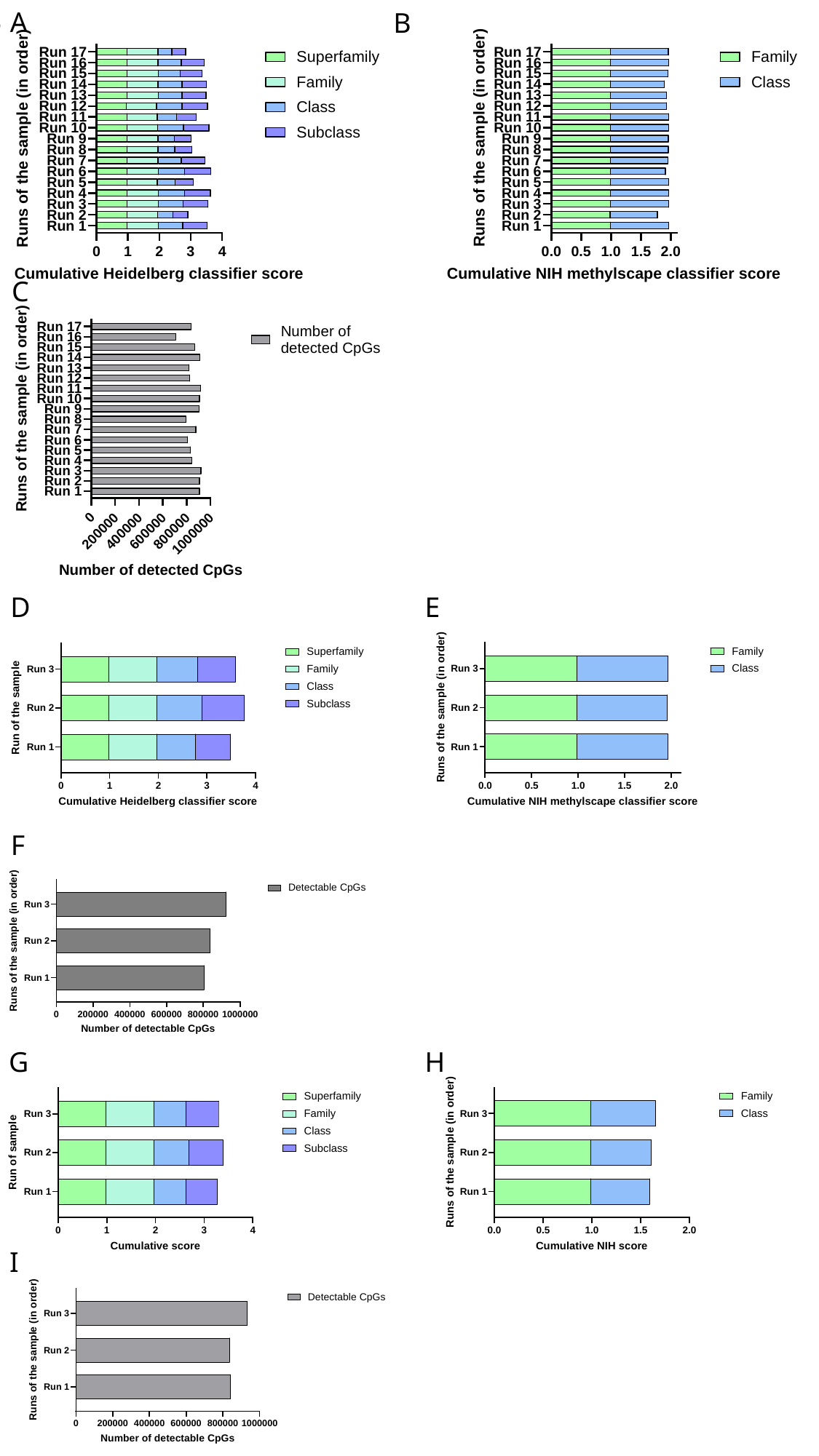

Supplementary Figure 5
A
B
C
D
E
F
G
H
I

## Slide 6
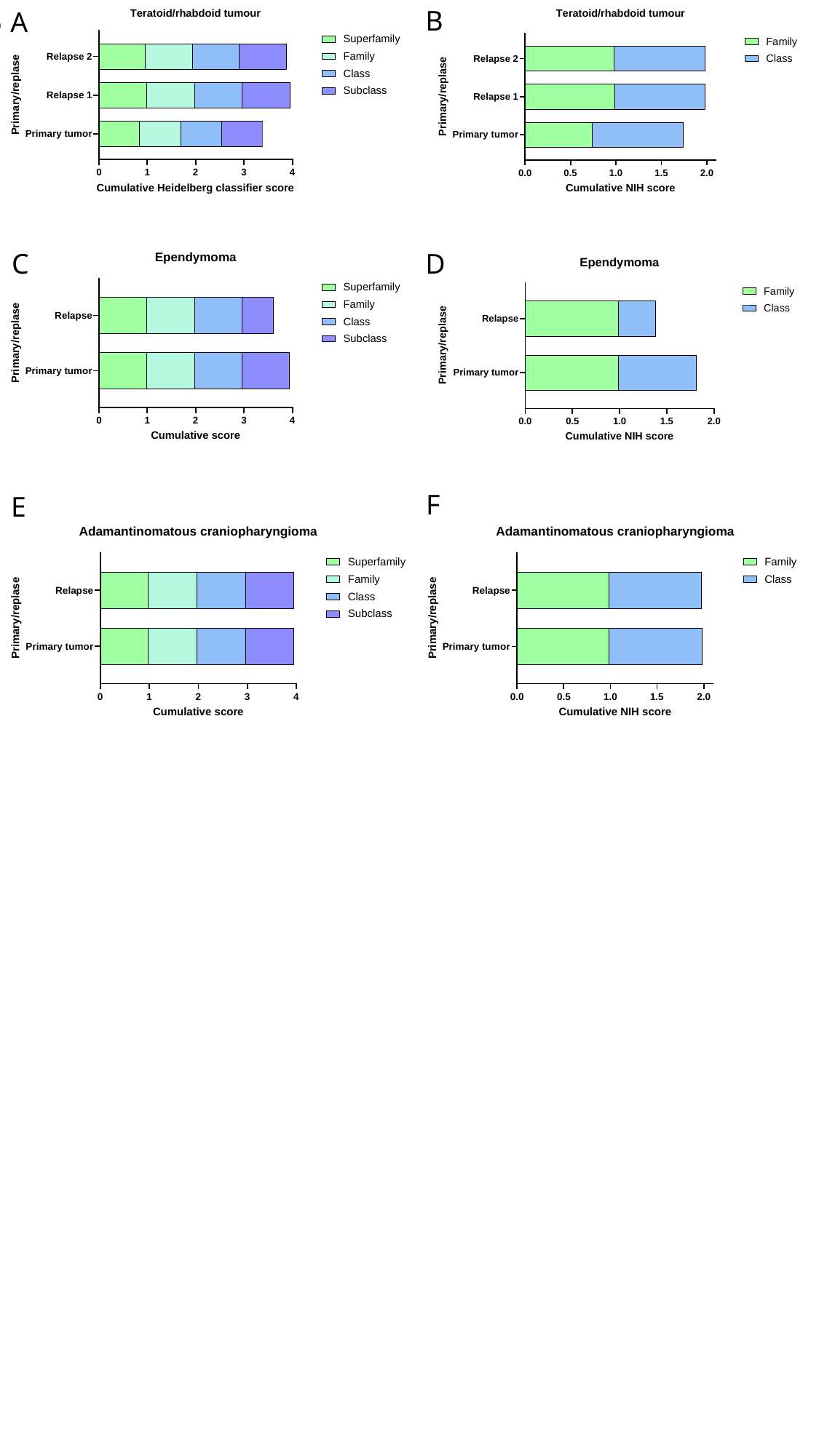

A
Supplementary Figure 6
B
C
D
F
E
